# Polyethylene wear rates in reverse total shoulder arthroplasty: a systematic review of biomechanical studies

**DOI:** 10.1016/j.xrrt.2025.07.020

**Published:** 2025-08-12

**Authors:** Sarang Agarwal, Ryan S. Ting, Rachel Farrelly, Bob Jang, John N. Trantalis

**Affiliations:** a Orthocentre Orthopaedic Research Institute, Sydney, Australia; b St George & Sutherland Clinical School, UNSW Sydney, Sydney, Australia; c Department of Orthopaedic Surgery, Concord Repatriation General Hospital, Sydney, Australia; d University of Sydney, Sydney, Australia

**Keywords:** Glenoid loosening, Highly cross-linked, HXLPE, Polyethylene wear, Reverse shoulder, Shoulder arthroplasty

## Abstract

**Background:**

Reverse total shoulder arthroplasty (rTSA) was classically indicated in rotator cuff deficient shoulders with osteoarthritis, although its indications and usage worldwide has grown in recent years. Loosening due to polyethylene wear is a significant concern, especially with increasing use of rTSA in younger patients, and as life expectancy increases. In the context of a lack of clinical data on polyethylene wear rates in rTSA, this study aimed to systematically review the biomechanical literature on factors affecting wear rates in rTSA, with comparison between highly cross-linked polyethylene (HXLPE) and non-HXLPE being of particular interest given the clinical differences shown in total hip arthroplasty.

**Methods:**

The PubMed, EMBASE, and Scopus databases were searched for biomechanical studies involving rTSA measuring polyethylene wear rates from April 4, 2024, to inception in accordance with the Preferred Reporting Items for Systematic Reviews and Meta-analyses guidelines.

**Results:**

The search returned 1,211 results, of which 380 were duplicates. Of the remaining 831 records, 773 were excluded based on title and 41 based on abstract, leaving 17 studies for full-text review. Nine biomechanical studies were included. One study showed that the wear rates were significantly lower in HXLPE humeral liners as compared to conventional polyethylene liners (36 mm glenosphere: HXLPE 36.5 ± 10 mm^3^/million cycles (MC) vs. ultra-high molecular weight polyethylene 83.6 ± 20.6 mm^3^/MC, *P* < .001). Wear rates were significantly higher in larger glenospheres (40 mm glenosphere 81.7 ± 23.9 mm^3^/MC vs. 32 mm glenosphere 68 ± 18.9 mm^3^/MC, *P* < .001) than in smaller glenospheres. There were no statistical differences in polyethylene wear rates between implants with vs. without a glenosphere with a central hole, rim damage to the humeral liner, inverse tribological pairing in the liner, and between retentive and nonretentive liners.

**Conclusion:**

HXLPE use in rTSA significantly decreases wear rate as compared to conventional polyethylene in a laboratory-controlled environment. Although this supports the superiority of the biomechanical properties of HXLPE, whether this translates to an improvement in clinical outcomes is undetermined, and limited by the lack of commercially available options despite the ubiquity of HXLPE in prostheses for other joints. Other factors associated with increased wear include larger glenospheres.

Arthroplasty of the glenohumeral joint is the third most common type of joint replacement following hip and knee arthroplasty.^26^ The modern reverse total shoulder arthroplasty (rTSA) was Food Drug Administration approved and became commercially available in 2003. Since then, the number of rTSA cases performed each year has increased exponentially. The Australian Orthopaedic Association National Joint Replacement Registry reported that a total of 49,230 cases were performed in 2022.^2^ Although, initially developed for cuff tear arthropathy, its indications have grown to include other conditions, such as massive irreparable rotator cuff tears and primary glenohumeral arthritis with intact rotator cuff tendons in the elderly.

Advances in knowledge and technology have meant that the rTSA has become an increasingly reliable procedure that produces predictable outcomes when paired with careful patient selection. Nevertheless, in addition to standard complications of arthroplasty, such as dislocation, infection, loosening, and periprosthetic fractures, complications specific to rTSA are of particular interest to shoulder surgeons.^23^^,^^24^

Wear, in tribological terms, refers to the progressive loss or displacement of material from a surface due to relative motion and contact with another surface, and is often measured via volumetric loss. In the context of arthroplasty, the generation of particulate debris can cause an inflammatory response that precipitates osteolysis.^14^ Wear, however, comprises several different entities, ranging from articulating surface wear, which may be caused by suprathreshold friction, difference in articulating materials, nonarticulating rim wear, which may be caused by edge-loading and micro-separation during movement, and surface defects caused by abutment.^6^^,^^14^

Scapular notching, as described by Sirveaux et al,^25^ is a complication of rTSA that is caused by bony erosion of the glenoid neck, which leads to glenoid bone loss and baseplate loosening and is a major complication of rTSA.^12^ Radiologically, component loosening can be defined by migration, tilt or shift of a prosthetic component, or by the presence of a complete radiolucent line at least 1.5 mm thick in the periprosthetic region.^5^ A recent meta-analysis of complications after rTSA found that the global scapular notching rate was 29.4% after a 3.5-year follow-up.^23^ A number of factors contribute to this undesirable evolution. Although evolution from the Grammont style rTSA to modern designs has led to significant improvements, with the introduction of eccentric glenospheres that inferiorize the metaglene/baseplate so the humeral tray can clear the scapula neck, polyethylene wear is still a significant problem. Scapular notching can lead to glenoid bone loss, and mechanical impingement is known to cause type 1 and 2 Nerot-Sirveaux notching.^30^ However, type 3 and 4 notching, which ultimately leads to baseplate failure, has been hypothesized to result from the inflammatory reaction due to polyethylene wear.^16^

The polyethylene humeral cup in ball-in-socket rTSA systems are liable to damage from articular surface wear at the interface with the glenosphere, and rim damage, which is most commonly observed at inferomedial edge in the context of scapular notching.^6^ Both of these processes generate polyethylene particles,^13^ which can contribute to osteolysis and ultimately aseptic loosening.

The use of highly cross-linked polyethylene (HXLPE) liners in total hip arthroplasty has resulted in significantly lower rates of osteolysis than conventional polyethylene liners clinically.^31^ HXLPE is a modified form of polyethylene that undergoes high dose irradiation, which results in a higher cross-link density that significantly improves the resistance to linear wear in comparison to conventional ultra-high molecular weight polyethylene (UHMWPE), which has fewer cross-linked chains.^28^

The benefit of HXLPE in rTSA, however, is undetermined. To our knowledge, there is a lack of clinical data on the influence of different types of polyethylene on wear rates in rTSA—the lack of HXLPE options in commercially available prostheses likely contributes to this phenomenon. Therefore the aim of the present study was to perform a systematic review of biomechanical studies to compare polyethylene wear rates between HXLPE and non-HXLPE liners and to identify design features that may help to reduce polyethylene wear rates.

## Materials and methods

### Search strategy

This review followed the Preferred Reporting Items for Systematic Reviews and Meta-Analyses guidelines. The protocol was registered in the International Prospective Register of Systematic Reviews (PROSPERO 2024 CRD42024528555). The search strategy was designed in consultation with a librarian.

The search was performed using the PubMed, EMBASE, and Scopus database on April 4, 2024, using the following search terms: (“Shoulder” OR “Glenohumeral”) AND (“Arthroplasty” OR “Replacement” OR “Prosthesis”) AND (“Polyethylene” OR “Highly cross-linked” OR “Cross-linked” OR “HXPLE” OR “XLPE”). Our search resulted in 1,211 titles. All publications were categorized using Endnote X10. Two reviewers (S.A. and R.S.T.) independently screened all available studies by title and abstract, then performed a formal full-text review of eligible studies. Disagreement between the 2 reviewers were resolved through discussion in consultation with a third reviewer acted as the arbiter (J.N.T.)

### Study selection criteria

Biomechanical studies investigating wear rates using polyethylene articulating surfaces in rTSA prostheses were eligible for inclusion. Studies that did not report on polyethylene wear rates, that were performed on anatomic total shoulder prostheses, clinical studies, computational analyses, case reports, commentaries, editorials, or had non-English full texts were excluded.

## Results

**Figure 1:**
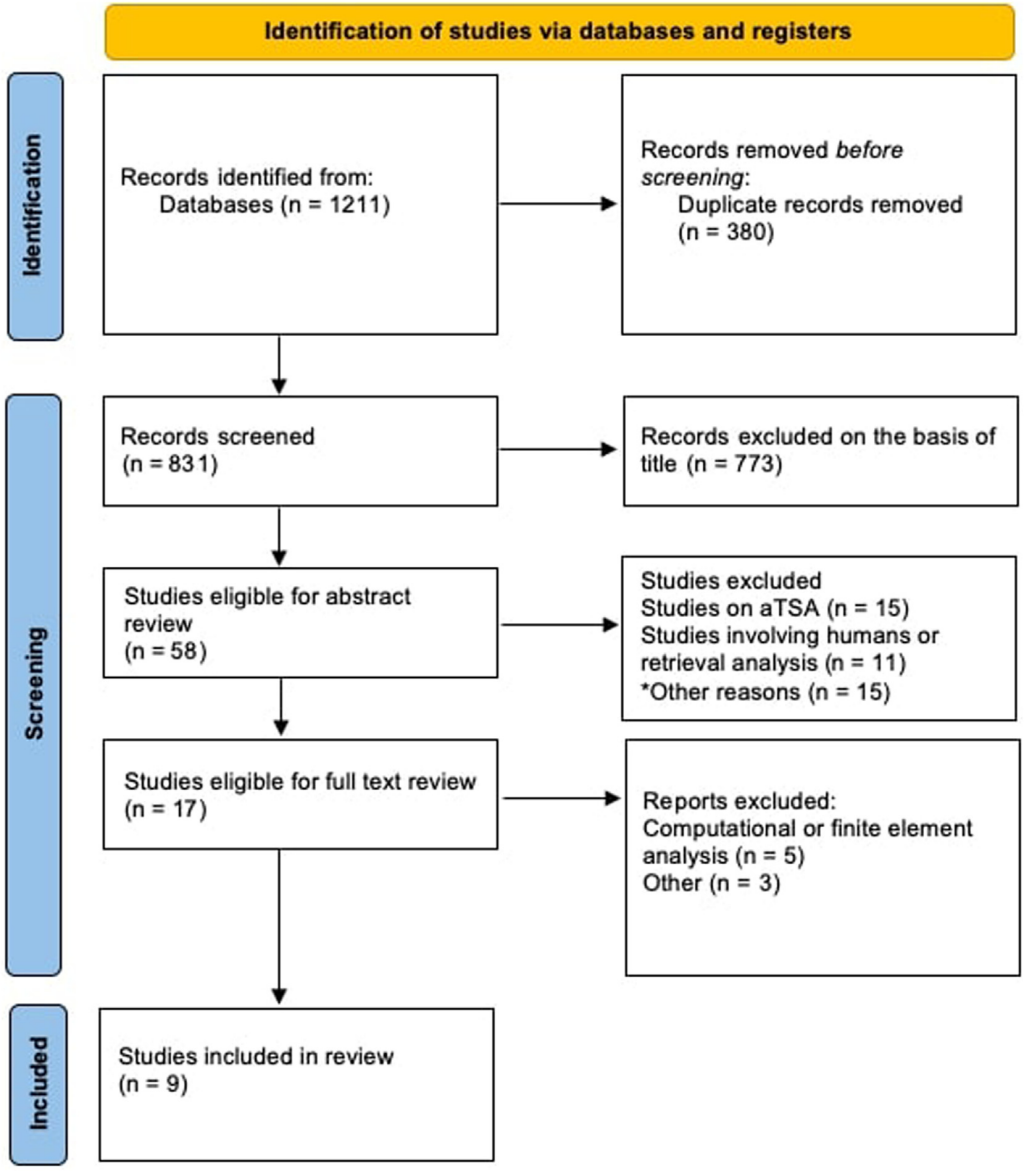
Preferred Reporting Items for Systematic Reviews and Meta-Analyses diagram for biomechanical studies looking at wear rates in reverse total shoulder arthroplasty. *aTSA*, anatomic total shoulder arthroplasty.

Our initial search yielded 1,211 articles. After removing duplicate titles, 831 records were screened for eligibility based on title and abstract, leaving 17 studies for full-text review. A total of 773 articles were excluded on the basis of title and 58 abstract reviews were conducted. Of those, 15 were excluded as they involved anatomic total shoulder replacement, 11 studies involved human subjects or retrieval analysis, 5 studies were computational analysis and 1 study was a conference abstract. This left 9 studies for inclusion (Fig. 1).

Six studies^4^^,^^8^^,^^9^^,^^15^^,^^17^^,^^27^ used multistation hip simulator for testing, whereas Kohut et al^11^ used 2 single station hip simulators for testing. Smith et al^26^ designed a 6-station shoulder simulator that was also used by Ramirez-Martinez et al^19^ All included studies cycled models through flexion-extension and adduction-abduction. Specimens taken for wear measurement were processed using different sera, and the contents and concentration of protein varied between studies. The number of cycles after which wear rates were measured ranged from 500,000 to 5 million cycles (MC), and wear measurements were taken at variable time points. The wear testing protocol and rates are summarized in Table I.

**Table I:** Comparison of wear rates results and previous conditions applied during reverse shoulders studies.

Study	Method of testing	Testing specimens	Summary of results (wear rates)
Vaupel et al^27^ (2012)	Multistation hip simulator [MTS Bionix]. Bovine serum with 21 g/L protein concentration. Motion and load: 20 - 617.82 N: Abd - Add 20 - 926.73 N: Flex. - Ext.	Custom UHMWPE humeral component with 36 mm CoCr femoral head as glenosphere: - with 5.0 mm central hole - without central hole	Glenosphere: - with 5.0 mm central hole: 126 mm3/MC - without central hole: 125 mm3/MC *P* value: 0.9 No significant difference in wear rates between above glenospheres
Carpenter et al^4^ (2015)	Multistation hip simulator [MTS Bionix]. Bovine serum with 21 g/L protein concentration. Motion and load: 20 - 618 N: Abd - Add 20 - 927N: Flex. - Ext.	Zimmer trabecular metal reverse shoulder system with 36 mm CoCr glenosphere and 36 mm diameter UHMWPE humeral liners: - retentive (12-degree elevation) - non-retentive (7-degree elevation)	- Retentive liners: 96.8 mm^3^/MC - Non-retentive liners: 88.1 mm^3^/MC *P* value = .076 No significant difference in wear rates between above humeral liners
Kohut et al^11^ (2012)	Two single station hip simulators [KUPA]. Bovine serum with 30 g/L protein concentration. Motion and load: 250 - 1000 N: Abd - Add, Flex. - Ext., Int. and Exr. rotation	Comparison between UHMWPE inlay with CoCr glenosphere and with inverse pairing	- UHMWPE glenosphere/CoCr inlay: 18.56 mg/MC - CoCr glenosphere/UHMWPE inlay: 13.21 mg/MC *P* value = N.A.
Smith et al^26^ (2015)	6 station shoulder simulator. “Newcastle shoulder simulator”. Newborn calf serum with 26 g/L protein concentration. Motion and load: 180 - 250 N: Abd - Add, Flex. - Ext., Int. and Exr. rotation	JRI Orthopaedics VAIOS reverse shoulder prosthesis with 42 mm CoCr glenosphere and 42 mm UHMWPE humeral liner	−14.3 mm^3^/MC
Griffith et al^8^ (2020)	Modified orbital bearing hip wear simulator [MATCO]. Non-iron alpha calf serum with 30 g/L protein concentration. Motion and load: 900 N peak load. Abd - Add, Flex. - Ext.	Delta XTEND 38 mm CoCr glenosphere with 38 mm UHMWPE humeral liner with 3 successive levels of damage in cups to simulate scapular notching.	- Average: 25.3 mm3/MC
Haggart et al^9^ (2017)	Multistation hip simulator [MTS Bionix]. Bovine serum with 21 g/L protein concentration. Motion and load: 20 - 618 N: Abd - Add 20 – 927 N: Flex. - Ext.	DJO Global reverse shoulder prosthesis with 32 mm and 40 mm CoCr glenosphere and matched UHMWPE humeral liners.	−40 mm glenospheres (mean: 81.7 +- 23.9 mm^3^/MC) −32 mm glenospheres (mean: 68 +- 18.9 mm^3^/MC) *P* value < .001 Wear rate of polyethylene liner with 40 mm glenosphere is significantly higher than that with 32 mm glenosphere
Peers et al^17^ (2015)	Multistation hip simulator [MTS Bionix]. Bovine serum with 21 g/L protein concentration. Motion and load: 20 - 617.8 N: Abd - Add 20 - 926.7 N: Flex. - Ext.	36 mm CoCr glenosphere (Zimmer) with custom 36 mm diameter UHMWPE and HXLPE humeral inserts.	- HXLPE liners: mean: 36.5 ± 10 mm^3^/MC - UHMWPE liners: mean: 83.6 ± 20.6 mm^3^/MC *P* value < .01 Wear rate of HXLPE is significantly less than that of UHMWPE
Langohr et al^15^ (2016)	Modified orbital bearing hip wear simulator (MATCO). Non-iron alpha calf serum 30 g/L protein concentration. Motion and load: 914 N peak load. Abd - Add, Flex. - Ext.	Delta XTEND 42 mm CoCr glenosphere with 42 mm UHMWPE humeral liner.	- Normal humeral liners: 42 mm3/MC - Notched humeral liners: 38 mm3/MC *P* value = N.A.
Ramirez-Martinez et al^19^ (2019)	6 station shoulder simulator. “Newcastle shoulder simulator”. Newborn calf serum with 26 g/L protein concentration. Motion and load: 180 -250N dynamic load: Flex. – Ext., Abd. – Add., Int. – Exr. rotation. 450N static load	JRI Orthopaedics VAIOS reverse shoulder prosthesis with 42 mm CoCr glenosphere and 42 mm UHMWPE humeral liner with 6.5 mm diameter hole in glenosphere.	−12 +- 3.9 mm^3^/MC *P* value: N.A.

*Flex*., flexion; *Ext*., extension; *Abd*., abduction; *Add*., adduction; *Int*., internal; *Exr*., external; *CoCr*, cobalt-chromium; *HXLPE*, highly cross-linked polyethylene; *UHMWPE*, ultra-high molecular weight polyethylene.

Smith et al^26^ found that the wear rate for UHMWPE was 14.3 mm^3^/MC after 4.5 MC using the Newcastle shoulder simulator. Langohr et al^15^ found that the mean wear rates for UHMWPE humeral liners was 42 mm^3^/MC vs. 38 mm^3^/MC (*P* = N.A.) for inferiorly notched UHMWPE liners using the modified orbital bearing hip wear simulator hip simulator.

### Conventional polyethylene vs. HXLPE

One study compared wear rates using different polyethylene humeral liners. Peers et al^17^ compared wear rates in conventional and HXLPE liners and observed that HXLPE exhibited a significantly lower wear rate (mean: 36.5 ± 10 mm^3^/MC) as compared to UHMWPE (mean: 83.6 ± 20.6 mm^3^/MC) (*P* < .01).

### Effect of glenosphere design

Three studies measured polyethylene wear rates with different designs of glenosphere. Vaupel et al^27^ compared UHMWPE with glenospheres with vs. glenospheres without holes in the articulating surface, and found that mean wear rates were 126 mm^3^/MC and 125 mm^3^/MC, respectively (*P* = .9). Ramirez-Martinez et al^19^ found that the mean wear rate of UHMWPE with a 42 mm glenosphere with a central hole was 12 +- 3.9 mm^3^/MC. When comparing wear rates in UHMWPE with 32 mm and 40 mm glenospheres, Haggart et al^9^ found significantly higher volumetric wear rates with 40 mm glenospheres (mean: 81.7 +- 23.9 mm^3^/MC) as compared to 32 mm glenospheres (mean: 68 +- 18.9 mm^3^/MC) at all time points (*P* < .001).

### Effect of humeral liner

Carpenter et al^4^ compared UHMWPE with nonretentive vs. retentive liners, and found that mean wear rates were 88.1 mm^3^/MC and 96.8 mm^3^/MC, respectively (*P* = .076). Griffiths et al^8^ compared wear rates in UHMWPE with normal cups versus those with fabricated rim damage that were intended to simulate rim damage after scapular notching. They measured wear rates using the modified orbital bearing hip wear simulator hip simulator after 3 successive blows with a rotary cutting tool. They found that after first and second blows, that there was a decrease in wear from the polyethylene insert. However, wear increased after the third simulated blow. The wear rates ranged between 24.1 mm3/MC and 26.1 mm3/MC over the course of testing and the average volumetric wear rate was 25.3 mm^3^/MC (*P* = N.A.).

### Effect of inversion of articulating bearing materials

Kohut et al^11^ presented the wear rates of UHMWPE with reverse pairing of prosthesis articular surfaces and found that the wear rate for UHMWPE glenosphere/cobalt-chromium inlay was 18.56 mg/MC compared to 13.21 mg/MC for cobalt-chromium glenosphere/UHMWPE inlay (*P* = N.A.).

## Discussion

The main objective of our review was to find out the difference in wear rates between conventional polyethylene and HXLPE articulating components in rTSA. In 1 biomechanical study, we found that the wear rates decrease significantly in HXLPE humeral liners as compared to conventional polyethylene liners. Although unrelated to the type of polyethylene, we also found that larger glenospheres also increase polyethylene wear rates, whereas retentive humeral liners, glenosphere with a central hole, rim damage to humeral liner, and inverse tribological pairing in liner and glenosphere did not seem to affect the wear rate after certain number motion cycles.

The most robust data on polyethylene wear can be found in hip arthroplasty.^10^^,^^18^^,^^21^^,^^22^ Hopper et al^10^ demonstrated that conventional crosslinked polyethylene had a wear rate of 0.17 mm/y compared with crosslinked polyethylene (XLPE) 0.03 mm/y. Notably, the osteolysis threshold is 0.1 mm/y. Yoon et al,^29^ in his meta-analysis comparing XLPE versus conventional polyethylene, concluded that XLPE significantly decreases the linear wear rate, overall osteolysis, and risk of overall wear-related revision in total hip arthroplasty. This contrasts with studies in knee arthroplasty, which do not show a significant difference between types of polyethylene liners.^28^

Clinical data in shoulder arthroplasty looking at the differences between polyethylene types are limited. We therefore undertook this meta-analysis to determine the difference in wear rates between conventional polyethylene and HXLPE in biomechanical studies. Biomechanical studies are not without their limitations. Firstly, we found that wear rates vary considerably between simulation environments. This could be due to different simulation devices, different patterns of wear, and different applied loads between studies. Furthermore, Desjardins et al^7^ showed that bovine calf serum with hyaluronic acid had been associated with higher wear rates, indicating that a different serum may also impact on results.

Of the biomechanical studies we assessed, 2 demonstrated a significant difference between polyethylene types. In the first study by Peers et al,^17^ UHMWPE was compared to HXLPE with regards to wear rates. They found that HXLPE produced 54% less wear particles than UHMWPE. They also found that HXLPE generated particles that were within the 0.1-1.0 μm phagocytable range and were less fibrillar, and therefore less inflammatory than those generated by UHMWPE wear.

Despite their being a limited number of biomechanical studies comparing types of polyethylene these studies still offer insight into rTSA designs which could affect polyethylene wear rates. These unique findings in each study with different implant philosophies, design, and biomechanical simulation methodologies may help improve future rTSA designs.

Metal glenospheres with holes reach a steady state of wear sooner than those without holes. This was determined in a biomechanical study by Vaupel et al^27^ where glenohumeral contact forces created by holding a 2 kg weight through various motions initially lead to a higher volumetric wear rate in glenospheres with holes. However, a ceiling effect was noted at 5 MC, after which a steady state was reached.

Larger diameter glenospheres, such as those used to restore deltoid tension and increase postoperative stability, may need to be weighed against potential for wear induced osteolysis. A biomechanical study by Haggart et al^9^ investigated the effect of glenosphere size on linear and volumetric wear rates. Linear wear refers to the depth of material lost in a specific vector during a motion cycle, whereas volumetric wear refers to the total volume of material lost during the motion cycle.^3^ They found that larger-sized glenospheres in rTSA had a significantly higher volumetric wear but less linear wear rates than smaller heads.^9^ This theory is further supported by hip literature, which reports a 61% increase in volumetric wear of HXLPE when the metallic femoral head size was increased from 22 mm to 36 mm.^1^

Retentive liners had a statistically significant greater total volume loss and higher volumetric wear rates at any specific time than nonretentive liners after 3.5 MC of testing.^4^ Initially the wear rates increased in both types liners; however, in later stages the difference became statistically nonsignificant after 3.6 MC, likely due to the increased contact area of retentive liners which result in a greater wear footprint.

The effect of successive liner rim damage was studied by wear testing of rTSA implants with reduced depth to radius ratio. It was inferred that the decreased contact from liner rim damage was counteracted by the simultaneous increase in contact pressure, resulting in more crosslinked polyethylene wear.^8^ This theory of increased contact pressure was further cemented by a second study using 100 station Super circularly translating pin-on-disk device resulting in a 6-fold increase in contact pressure on UHMWPE which increased the wear rate by 2.6-fold.^21^

The final component design we consider is inversed tribological pairing. Inverted bearing rTSA avoids abutment of polyethylene against the scapula, thus preventing biological notching.^11^ Ranieri et al^20^ reported a 98.3% revision-free survival at 10 years in a series of inverted bearing rTSA and a lower scapular notching incidence in their series compared with the literature. They found that the occurrence of type 1 and 2 notching was likely to be mechanically induced. Notably, type 3 and 4 notching as described by Nerot-Sirveaux grade cannot be explained by biomechanical mechanisms.^25^ In conclusion particle generation, inflammation, and polyethylene particle-induced osteolysis may be an explanation for the failure in noninversed tribological pairing.

This study had several limitations. First of all, biomechanical studies do not replicate end-of-range abutment conditions and therefore may underestimate the production of large wear particles and their contribution to volumetric wear. In hemispherical glenosphere designs, articular play can occur in adduction and at the extremes of rotation. This may result in point contact overload, leading to localized damage to the polyethylene liner—a phenomenon not addressed in this study. Although biomechanical studies enhance our understanding of wear mechanisms, future research should aim to compare multiple factors within a unified and consistent experimental setup to draw more comprehensive conclusions. We mentioned the considerably different biomechanical simulation environments which makes it difficult to directly compare. Furthermore, the small number of studies making a direct comparison of polyethylene types limits the effect of the results. The available biomechanical studies in rTSA point at a myriad of design considerations, considerations that need to be further substantiated by clinical studies with long-term follow-up. Overall, despite the limitations, biomechanical studies have their place by allowing the extremes, such as increased contact pressure and millions of repetitions to be tested which may not be possible in clinical studies.

## Conclusion

This review found that use of HXLPE liners and smaller glenospheres (32 mm) resulted in significantly less wear than when conventional polyethylene liners or larger glenospheres (40 mm) were used in rTSA, with no statistical difference in wear rates between implants with vs. without a glenosphere with a central hole, rim damage to the humeral liner, inverse tribological pairing in the liner, and between retentive and nonretentive liners. With all the emphasis on optimization of the surgical technique and implant technology, it is hard to understand why there is a lack of commercially available HXLPE options in rTSA, especially given that HXLPE has proven to be a superior material to conventional polyethylene, in addition to the ubiquity of HXLPE options in hip and knee arthroplasty. Whether the biomechanical superiority of HXLPE in rTSA translates clinically in the form of reduced polyethylene-induced osteolysis and the associated complications, however, remains undetermined.

## Disclaimers:

Funding: No funding was disclosed by the authors.

Conflicts of interest: The authors, their immediate families, and any research foundation with which they are affiliated did not receive any financial payments or other benefits from any commercial entity related to the subject of this article.
